# A Synthetic Biology Approach for a Vaccine Platform against Known and Newly Emerging Serotypes of Bluetongue Virus

**DOI:** 10.1128/JVI.02183-14

**Published:** 2014-11

**Authors:** Sandro Filipe Nunes, Claude Hamers, Maxime Ratinier, Andrew Shaw, Sylvie Brunet, Pascal Hudelet, Massimo Palmarini

**Affiliations:** a MRC–University of Glasgow Centre for Virus Research, Glasgow, United Kingdom; b Merial Animal Health, Lyon, France

## Abstract

Bluetongue is one of the major infectious diseases of ruminants and is caused by bluetongue virus (BTV), an arbovirus existing in nature in at least 26 distinct serotypes. Here, we describe the development of a vaccine platform for BTV. The advent of synthetic biology approaches and the development of reverse genetics systems has allowed the rapid and reliable design and production of pathogen genomes which can be subsequently manipulated for vaccine production. We describe BTV vaccines based on “synthetic” viruses in which the outer core proteins of different BTV serotypes are incorporated into a common tissue-culture-adapted backbone. As a means of validation for this approach, we selected two BTV-8 synthetic reassortants and demonstrated their ability to protect sheep against virulent BTV-8 challenge. In addition to further highlight the possibilities of genome manipulation for vaccine production, we also designed and rescued a synthetic BTV chimera containing a VP2 protein, including regions derived from both BTV-1 and BTV-8. Interestingly, while the parental viruses were neutralized only by homologous antisera, the chimeric proteins could be neutralized by both BTV-1 and BTV-8 antisera. These data suggest that neutralizing epitopes are present in different areas of the BTV VP2 and likely “bivalent” strains eliciting neutralizing antibodies for multiple strains can be obtained.

**IMPORTANCE** Overall, this vaccine platform can significantly reduce the time taken from the identification of new BTV strains to the development and production of new vaccines, since the viral genomes of these viruses can be entirely synthesized *in vitro*. In addition, these vaccines can be brought quickly into the market because they alter the approach, but not the final product, of existing commercial products.

## INTRODUCTION

Bluetongue is a major infectious disease of ruminants and is caused by bluetongue virus (BTV), an arbovirus in the Reoviridae family transmitted from infected to uninfected mammalian hosts by Culicoides biting midges (1, 2).

BTV has a genome composed of 10 linear double-stranded RNA segments encoding seven structural and four nonstructural proteins (3–5). The BTV virion is an icosahedral particle assembled as a triple-layered capsid (6, 7). There are at least 26 BTV serotypes (BTV-1 to BTV-26) circulating worldwide. Serotypes are determined primarily by differences in the outer capsid protein VP2, which mediates viral entry into the cell and is the target for neutralizing antibodies in infected animals (8–13). VP2 and, to a lesser extent, VP5 interact with the VP7 protein, the major component of the underlying core (14).

BTV infection in mammalian hosts results in inapparent to severe clinical symptoms generally associated with damage to small blood vessels (2, 15, 16). Serotype-specific neutralizing antibodies are generated upon infection in both naturally or experimentally infected ruminants and provide little or no protection against heterologous serotypes (17, 18).

Traditionally, regions where bluetongue is endemic have been limited to tropical and subtropical areas of the world (19, 20). However, in the last 15 years, similarly to some other arbovirus infections, bluetongue has expanded its geographical limits. Since 1998, outbreaks caused by various BTV serotypes have been increasingly observed in Northern Africa and Europe (21–23).

Vaccination of susceptible livestock remains the most effective strategy for the control of BTV epidemics. Currently, only two different types of vaccines are available commercially: live attenuated vaccines, traditionally obtained from the successive passage of BTV in embryonated eggs or tissue culture, and inactivated whole-virus vaccines, in which BTV viruses are grown in tissue culture and later chemically inactivated.

Live attenuated vaccines have been used for decades in South Africa where bluetongue is endemic (24). These vaccines elicit strong neutralizing antibody and likely cell-mediated immune responses and confer long-term protection against homologous BTV infection (18). However, their use in Southern Europe, although effective in most cases, has been a cause of concern as some strains have been proven to be (i) poorly attenuated, (ii) teratogenic and affecting pregnancy, (iii) transmitted to nonvaccinated animals, and (iv) reassorted with wild-type viruses (25–29). For these reasons, the use of live attenuated viruses for BTV control in Europe was discontinued, and several vaccine manufacturers developed whole-virus inactivated vaccines (18, 30). These vaccines were proven to protect vaccinated animals against homologous BTV challenge. Although the duration of immunity induced by inactivated vaccines is shorter compared to that induced by live vaccines, their use helped to control and eventually eliminate BTV-1 and BTV-8 from Central and Northern Europe (18, 31–33).

The advent of synthetic biology approaches and the development of reverse genetics systems has allowed the rapid and reliable design and production of pathogen genomes which can be subsequently manipulated for vaccine production. In the present study, we describe the development of a strategy for the design and production of inactivated BTV vaccines that can significantly reduce the time taken from the identification of a new BTV emerging strain to the development and production of a new vaccine.

## MATERIALS AND METHODS

### Cells.

BSR, Vero, and BHK_21_ cells were grown in Dulbecco modified Eagle medium (DMEM) supplemented with 5% fetal bovine serum. Cells were incubated at 35 or 37°C, depending on the experimental setting, in a humidified incubator with 5% CO_2_. BSR cells were used for the recovery of “synthetic” BTV (sBTV) reassortants (5, 30). BHK21 cells were used for the production of BTV reassortants according to industry practice (20, 32), while Vero cells are cells recommended by the OIE (World Animal Health Organization) for the diagnosis of BTV infection in ruminants by seroneutralization assays (34).

### Plasmids.

Plasmids used for the rescue of BTV-1 of BTV-8 by reverse genetics were described previously (4). Similar plasmids containing segment 2 (encoding VP2) or segment 6 (encoding VP5) of the reference strains of BTV-2 to -24 and the recently described BTV-25 and -26 were synthesized commercially (Genscript) (Table 1). In addition, a chimeric BTV-1/BTV-8 segment 2 was also synthesized in which the nucleotide sequence encoding amino acid residues 220 to 429 of the BTV-8 VP2 were introduced into the homologous region of the BTV-1 segment 2.

**TABLE 1:** GenBank accession numbers of the BTV viral segments used in this study

Serotype	GenBank accession no.		
	Segment 2	Segment 6	BTV-1 backbone
BTV-1	FJ969720	FJ969723	
BTV-2	AJ585123	JN255867	Segment 1, FJ969719
BTV-3	AJ585124	AJ586697	
BTV-4	AJ585125	AJ586699	
BTV-5	AJ585126	AJ586700	Segment 3, FJ969721
BTV-6	AJ585127	AJ586703	
BTV-7	AJ585128	AJ586704	
BTV-8	AM498052	AM498056	Segment 4, FJ969722
BTV-9	AJ585130	AJ586708	
BTV-10	AJ585131	AJ586709	
BTV-11	AJ585132	AJ586710	Segment 5, FJ969724
BTV-12	AJ585133	AJ586711	
BTV-13	AJ585134	AJ586713	
BTV-14	AJ585135	AJ586714	Segment 7, FJ969725
BTV-15	AJ585136	AJ586716	
BTV-16	AJ585137	AJ586719	
BTV-17	AJ585138	AJ586720	Segment 8, FJ969726
BTV-18	AJ585139	AJ586721	
BTV-18	AJ585140	AJ586722	
BTV-20	AJ585141	AJ586723	Segment 9, JN848767
BTV-21	AJ585142	AJ586724	
BTV-22	AJ585143	AJ586725	
BTV-23	AJ585144	AJ586727	
BTV-24	AJ585145	AJ586730	
BTV-25	EU839840	EU839842	
BTV-26	HM590642	JN255159	Segment 10, FJ969728

### Recovery of sBTV reassortants.

sBTV reassortants were rescued *in vitro* by reverse genetics as previously described (4, 35). Briefly, DNA plasmids containing the eight genomic segments of the BTV-1 backbone, and each of the segments 2 and 6 of the 26 different BTV serotypes were linearized with restriction enzymes and purified by phenol-chloroform extraction. The linearized plasmids were then used as the templates for the *in vitro* synthesis of capped BTV-like RNA using mMESSAGE mMACHINE T7 Ultra kit (Ambion) according to the manufacturer's instructions. RNA was then extracted by phenol-chloroform and further purified by using an RNeasy minikit (Qiagen). For the rescue of synthetic BTV virus, monolayers of BSR cells in 12-well plates were transfected twice with BTV RNAs using Lipofectamine 2000. First, 1 × 10^11^ RNA molecules of each segment encoding VP1, VP3, VP4, NS1, VP6, and NS2 were diluted in Opti-MEM I reduced serum medium containing 0.5U/μl of RNAsin Plus (Promega) for 5 min and mixed with Lipofectamine 2000 diluted in Opti-MEM I reduced serum medium. After 25 min of incubation at room temperature, the mixture was added dropwise to the cells and transferred to a humidified incubator at 35°C. After 16 to 18 h, a second transfection was carried out with 1 × 10^11^ molecules of all the BTV segments. sBTVs were plaque purified from supernatants of transfected cultures displaying a cytopathic effect. Viruses obtained by reverse genetics based on the reference strain of BTV-1 from South Africa and the European BTV-8 strain (NET2006/04) were described previously (4). Each sBTV reassortant is formed by a “backbone” (i.e., all the nonstructural and enzymatic proteins in addition to VP7 and VP3) of BTV-1 and an outer capsid layer comprising VP2 and VP5 (or only the VP2) of a heterologous serotype. Each synthetic virus is referred in this study with “B1” (indicating the backbone of BTV-1), followed by the serotype of the VP2 (encoded by Seg-2) and VP5 (encoded by Seg-6) combination used. For example, _B1_BTV-8^VP2/VP5^ is a synthetic virus containing the backbone of BTV-1 with the VP2 and VP5 of BTV-8. Synthetic reassortants with a heterologous VP2-VP5 combination have an additional number referring to the serotype from which the VP5 was obtained. For example, _B1_BTV-24^VP2(4-VP5)^ has the backbone of BTV-1, the VP2 from BTV-24, and the VP5 from BTV-4. An additional synthetic BTV was rescued incorporating the chimeric BTV-1/BTV-8 VP2 segment and was designated _B1_BTV-1^(8-VP2-220-429)^.

### sBTV plaque phenotype and replication kinetics.

Viral plaque phenotypes of each sBTV reassortant were assessed in Vero cells. Cell monolayers were infected with 10-fold viral dilutions in DMEM for 2 h at 37°C, after which media containing the virus was removed, and the cells were washed with phosphate-buffered saline, followed by incubation with 3 ml of medium containing 1.2% Avicel (FMC Biopolymer) at 37°C for 72 h. The plates were fixed with 4% formaldehyde for 1 h. The monolayer was incubated with a primary rabbit antibody against the BTV VP7 protein (4), followed by an anti-rabbit horseradish peroxidase (HRP)-conjugated secondary antibody, and finally stained with a TrueBlue peroxidase staining kit (KPL). The replication kinetics of each synthetic virus was assessed in BHK_21_ cells as already described (4) using a multiplicity of infection (MOI) of 0.001.

### Inactivated vaccine production.

_B1_BTV-8^VP2/VP5^ and _B1_BTV-8^VP2^ were grown for 48 h in BHK_21_ cells in 50-liter bioreactors at 37°C in 5% CO_2_ humidified atmosphere at the Merial S.A.S. laboratory according to standard industrial procedures. Prior to inactivation, virus cultures were treated with chloroform, mechanically homogenized with an Ultra-Turrax T50 (IKA), and clarified by filtration and centrifugation. Formaldehyde (0.5 mg/ml) was then added to the filtered supernatant, which was subsequently treated twice with binary ethyleneimine (1.5 mM). This inactivation step was carried out at 37°C for 24 h. Complete inactivation of the virus was confirmed by serially passaging the treated supernatants three times in Vero cells. The inactivated supernatant was filtered in high-flow-capacity filters, concentrated twice, and purified by immobilized metal ion affinity chromatography. The antigen dosage was then measured by using dot blot analysis of the VP2 protein in parallel with an enzyme-linked immunosorbent assay for the VP7 protein in order to use amounts of antigen identical to the commercial BTVPUR AlSap vaccines (30). Vaccines were blended with aluminum hydroxide and saponin.

### Vaccination and BTV challenge.

All animal experiments were carried out in accordance with EU legislation and conducted in a high-containment animal facility. Lacaune crossbred sheep (4 to 5 months old), confirmed to be seronegative for BTV-8 by serum neutralization at the beginning of the study, were divided into four groups: group 1 (G1; *n* = 6) was the unvaccinated control group, G2 (*n* = 5) was vaccinated with _B1_BTV-8^VP2/VP5^, G3 (*n* = 5) was vaccinated with _B1_BTV-8^VP2^, and G4 (*n* = 5) was vaccinated with a low antigen dose (1/10 compared to the dose used for G3) of _B1_BTV-8^VP2^. Sheep were vaccinated subcutaneously with a single dose of 1 ml of each sBTV vaccine, while control sheep were not vaccinated.

At 21 days after vaccination, individual rectal temperatures were recorded, and serum samples were collected before sheep were challenged intradermally with 3 ml of a virulent North European BTV-8 strain (10^7^ genome copy numbers/ml) distributed among approximately 30 separate injection points. Sheep were monitored daily up to 14 days postchallenge for rectal temperature and clinical signs (general and body condition, congestion and/or edema, hypersalivation, nasal discharge/crusts, plaintive bleating, swollen lymph nodes, locomotion difficulty, and respiratory and digestive problems). A clinical score was assigned for the following signs: between 0 and 3 for general condition, between 0 and 1 for body condition, and between 0 and 4 for hyperthermia defined as body temperature above 40°C. Any other clinical signs scored 1 if present. The individual scores for each sheep were added together, resulting in a daily clinical score. Blood samples were collected on days 5, 7, 9, 12, and 14 postchallenge in order to detect BTV RNA as an indication of viremia by quantitative reverse transcription-PCR (RT-PCR) (see below). Neutralizing antibody titers in the serum of each animal were also measured at day 14 postchallenge (see below).

### Neutralization assays.

The presence of neutralizing antibodies in vaccinated and control sheep was assessed by microneutralization assays as already described (30). Sera were collected at the beginning of the study before vaccination (day −21), at the time of BTV-8 challenge (day 0), and 2 weeks postchallenge (day 14). The 50% protective dose (PD_50_) for each serum sample, defined as the serum dilution that inhibits BTV infection in 50% of Vero cell cultures, was then determined by using a linear regression after angular transformation. Samples below the detection limit of 0.48 log_10_ PD_50_ were considered negative.

### Quantitative RT-PCR.

RNA was extracted from blood samples by using a QIAamp viral RNA minikit (Qiagen) according to the manufacturer's instructions. One-step quantitative RT-PCR for the amplification of a conserved region of segment 10 was performed using TaqMan EZ RT-PCR core reagent (Applied Biosystems). Serial 10-fold dilutions of RNA standard *in vitro* transcribed from the BTV segment 10 were used to obtain a standard curve.

## RESULTS

### Rescue of sBTV viruses.

We obtained DNA plasmids containing commercially synthesized genomic segments encoding VP2 and VP5 (outer core proteins) of the reference strains of BTV-1 to -26. Plasmids were designed in order to be used in BTV rescue experiments as already described (4, 35). Initially, cells were transfected using homologous Seg-2 and Seg-6 from each serotype, in addition to plasmids containing genomic segments forming the “backbone” of the synthetic viruses (Seg-1, -3, -4, -5, -7, -8, -9, and -10) encoding the remaining structural, enzymatic, and nonstructural proteins from BTV-1. This strategy was successful for the rescue of 16 different BTV serotypes, resulting in the generation of the following synthetic BTV viruses: _B1_BTV-1^VP2/VP5^, _B1_BTV-2^VP2/VP5^, _B1_BTV-3^VP2/VP5^, _B1_BTV-4^VP2/VP5^, _B1_BTV-6^VP2/VP5^, _B1_BTV-8^VP2/VP5^, _B1_BTV-9^VP2/VP5^, _B1_BTV-11^VP2/VP5^, _B1_BTV-13^VP2/VP5^, _B1_BTV-17^VP2/VP5^, _B1_BTV-20^VP2/VP5^, _B1_BTV-21^VP2/VP5^, _B1_BTV-22^VP2/VP5^, _B1_BTV-23^VP2/VP5^, _B1_BTV-25^VP2/VP5^, and _B1_BTV-26^VP2/VP5^. Seg-2 and Seg-6 of BTV-25 were synthesized using the untranslated regions of the homologous segments of BTV-1.

For synthetic viruses that we were not able to rescue using homologous VP2 and VP5, we attempted rescue experiments using each Seg-2 (encoding VP2) in combination with the remaining 25 heterologous Seg-6 (encoding VP5). Following this approach, two new sBTV were generated: _B1_BTV-14^VP2(6-VP5)^, rescued using the VP2 of BTV-14 and the VP5 of BTV-6, and _B1_BTV-24^VP2(4-VP5)^, rescued using the VP2 of BTV-24 and the VP5 of BTV-4. Other strategies used to rescue the synthetic viruses with the outer core proteins of BTV-5, BTV-7, BTV-10, BTV-12, BTV-15, BTV-16, BTV-18, and BTV-19 included the use of Seg-2 with the untranslated region of BTV-1 and the use of homologous VP2, VP5, and VP7. In none of these cases were we able to rescue stable viruses that could be propagated beyond one passage after transfection, suggesting that infectious viruses were generated but that they were unstable (data not shown). We sequenced a portion of segments 2 and 6 for all of the reassortants successfully rescued to confirm the identity of each virus produced. In addition, we repeated each rescue at least three times independently (using two different plasmid preparations) in order to rule out that the failure in rescuing certain reassortants was due to deleterious mutations arising by chance during the rescue experiments.

### Replication kinetics of synthetic viruses.

**FIG 1:**
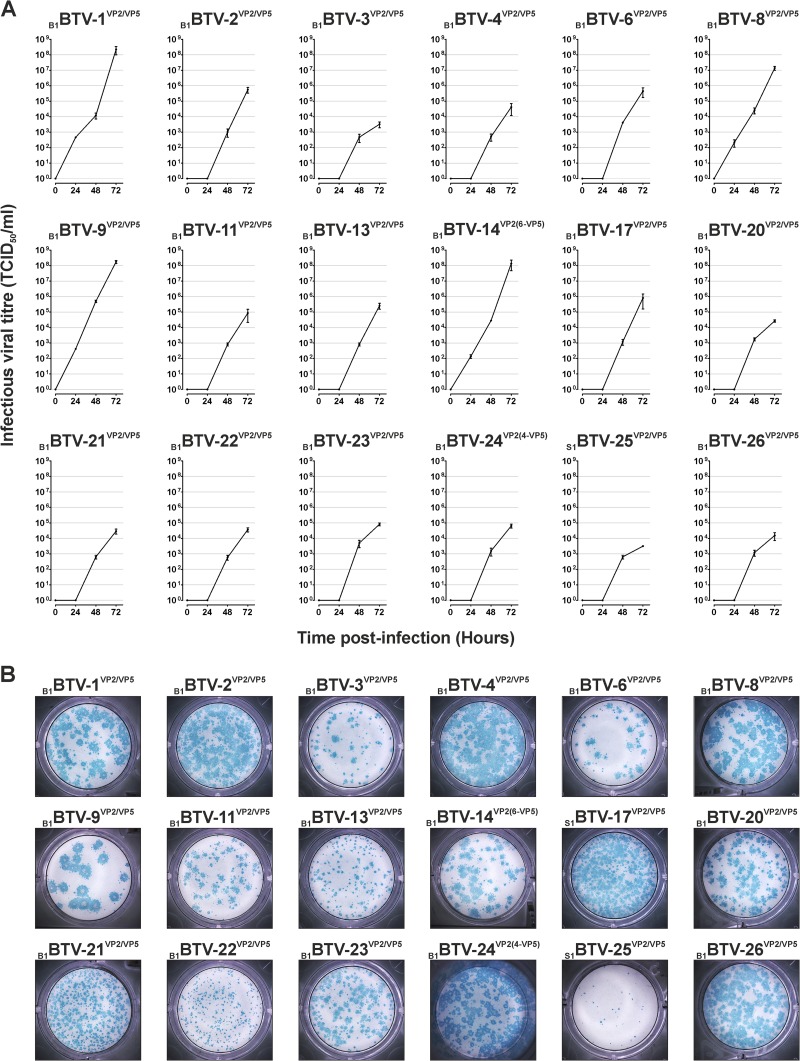
Replication kinetics and plaque phenotype of synthetic viruses. (A) Viral replication kinetics of synthetic viruses in BHK_21_ cells. Confluent monolayers were infected with each sBTV reassortant at an MOI of 0.001, and the virus supernatants were titrated as described in Materials and Methods. (B) Morphology of plaques induced by synthetic viruses in Vero cells. Immunostaining of viral plaques was performed by using a BTV-1 VP7 antibody, followed by an HRP-conjugated secondary antibody and staining with TrueBlue.

Replication kinetics of the rescued synthetic viruses were assessed in BHK_21_ cells using a low MOI (0.001) in order to simulate the initial production of a viral master stock for vaccine production (Fig. 1A). A total of 14 of the 16 synthetic viruses released virus into the supernatant of infected cells, reaching titers higher than 10^4^ TCID_50_ per ml at 72 h postinfection (p.i.). Differences in the replication kinetics between viruses reaching titers of >10^6^ TCID_50_/ml at 72 h p.i. (e.g., _B1_BTV-1^VP2-P5^, _B1_BTV-9^VP2/VP5^) and those with lower titers (<10^6^ TCID_50_/ml) were especially evident in the first 24 h. Indeed, the titers of the three reassortants with lower yields at 72 h p.i. (_B1_BTV-2^VP2/VP5^, _B1_BTV-25^VP2/^VP5, and _B1_BTV-26^VP2/VP5^) increased 10- to 1,000-fold by 120 h p.i., reaching levels compatible with the other reassortants produced in this study (data not shown). The size of the plaques induced in VERO cells appeared to be largely, but not always, correlated to the titers reached in replication kinetic assays in BHK_21_ cells (Fig. 1B).

### Vaccination with inactivated synthetic BTV-based vaccines incorporating the BTV-8 VP2 protein confers protection against homologous challenge.

**FIG 2:**
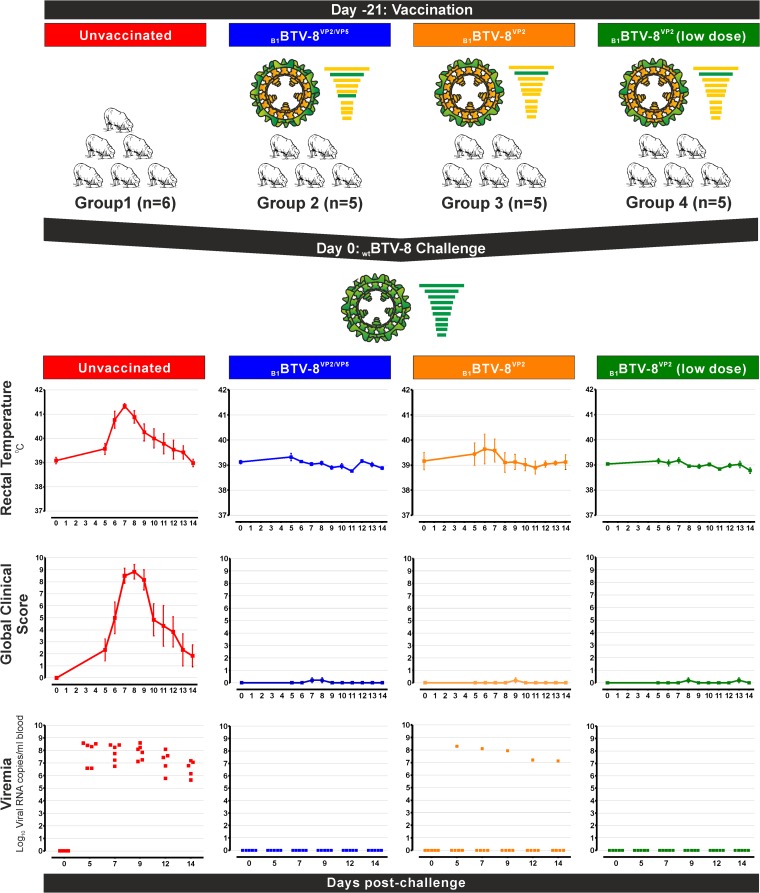
Sheep vaccinated with _B1_BTV-8^VP2/VP5^ and _B1_BTV-8^VP2^ are protected against wild-type BTV-8 challenge. Vaccine trials were carried out using four groups of Lacaune crossbred sheep. Group 1 animals were not vaccinated, while groups 2 and 3 were vaccinated with either _B1_BTV-8^VP2/VP5^ and _B1_BTV-8^VP2^, respectively. An additional group of sheep was also vaccinated with _B1_BTV-8^VP2^ (group 4) but with a lower vaccine dose than that used in current vaccine formulations. At 21 days postvaccination, each group was challenged with a virulent BTV-8 strain, and each animal was assessed for the onset of fever, clinical signs, and viremia. A total of 14 of 15 vaccinated animals in groups 2 to 4 were protected against BTV-8 challenge. One animal in group 3 developed fever and displayed viremia.

In order to validate the use of synthetic viruses as inactivated vaccines against BTV, we carried out vaccination trials using _B1_BTV-8^VP2/VP5^ and _B1_BTV-8^VP2^ (Fig. 2). These strains were inactivated with binary ethyleneimine and prepared in an industrial setting at the Merial S.A.S. laboratory similarly to the existing inactivated commercial vaccines (30).

For the vaccine trial, sheep were assigned into four groups: an unvaccinated control group (G1) and three other groups in which animals were inoculated with vaccine preparations containing identical antigen payload dose to commercial vaccines of _B1_BTV-8^VP2/VP5^ (G2) and _B1_BTV-8^VP2^ (G3) and a lower antigen dose (1/10) of _B1_BTV-8^VP2^ (G4). Animals were subsequently challenged intradermally with a virulent BTV-8 strain (Fig. 2 and Table 2).

**TABLE 2:** BTV-8 neutralizing antibody response in vaccinated and control sheep

Group	Animal	BTV-8 neutralizing antibody titer^*a*^ at:		
		Day −21	Day 0	Day 14
G1 (unvaccinated control)	10349	<0.48	<0.48	0.89
	10372	<0.48	<0.48	1.12
	10375	<0.48	<0.48	2.08
	10412	<0.48	<0.48	1.28
	10434	<0.48	<0.48	n.a.
	20133	<0.48	<0.48	1.20
	Mean ± SD	<0.48	<0.48	1.31 ± 0.45
G2 (_B1_BTV-8^VP2/VP5^)	10358	<0.48	0.53	2.08
	10376	<0.48	0.80	2.08
	10379	<0.48	0.72	1.76
	10414	<0.48	1.20	1.20
	20152	<0.48	0.72	1.76
	Mean ± SD	<0.48	0.79 ± 0.25	1.78 ± 0.36
G3 (_B1_BTV-8^VP2^)	10348	<0.48	0.72	1.68
	10360	<0.48	0.80	1.85
	10371	<0.48	<0.48	1.76
	10374	<0.48	<0.48	2.16
	10424	<0.48	0.53	1.76
	Mean ± SD	<0.48	<0.60 ± 0.15	1.84 ± 0.19
G4 (_B1_BTV-8^8VP2^, low dose)	10356	<0.48	<0.48	1.77
	10363	<0.48	<0.48	2.64
	10385	<0.48	0.72	1.20
	10420	<0.48	<0.48	1.37
	20103	<0.48	<0.48	1.76
	Mean ± SD	<0.48	<0.53 ± 0.11	1.75 ± 0.56

a The neutralizing titer in each sheep was expressed as log_10_ of the 50% protective dose (PD_50_).

As anticipated, all six unvaccinated control sheep (G1) showed fever and developed clinical signs commonly associated with BTV infection. The peak of fever was reached at day 7 postchallenge (41.3 ± 0.2°C) in which all animals exhibited congestion of the ears, eyes, nostrils, and lips, edema in the lips, and erythema (redness of skin). Due to the severity of the clinical signs, one sheep had to be euthanized at day 10 postchallenge. Viremia was detected throughout the 14 days in all animals of the group. In sheep vaccinated with _B1_BTV-8^VP2/VP5^ (G2) no increase in rectal temperature was observed in any of the vaccinated animals, and only mild respiratory signs were observed in one animal on days 7 and 8 postchallenge. Viremia was not detected in any of the animals of this group.

_B1_BTV-8^VP2^ was used in the vaccination of groups G3 and G4. In both vaccination groups, the presence of BTV-8 VP2 protein prevented fever and viremia. However, one animal in G3 did not seem to respond to vaccination and exhibited mild clinical signs of infection. Viremia was observed in this sheep to similar levels observed in the unvaccinated controls. Interestingly, 4 of the 5 animals in G4 did not develop neutralizing antibodies after vaccination but were protected against virulent BTV challenge. These data reinforce the idea that the presence of detectable neutralizing antibodies in vaccinated animal is not absolutely required for protection against BTV infection.

### Synthetic BTVs with a chimeric BTV-1 and BTV-8 VP2 are neutralized by both BTV-1 and BTV-8 antisera.

**FIG 3:**
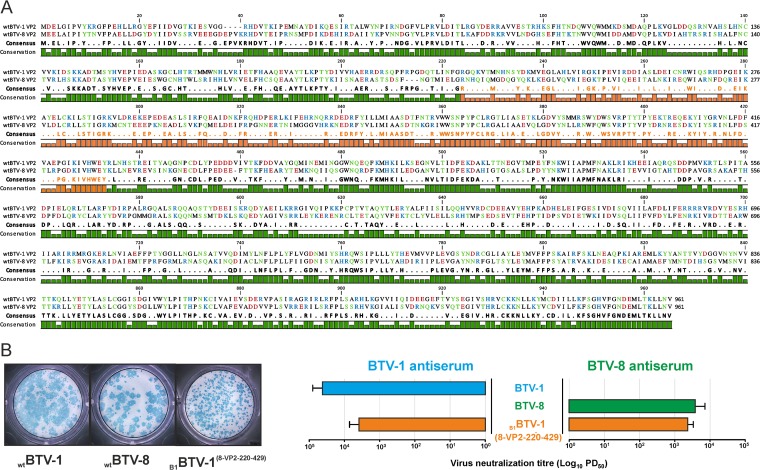
A synthetic virus with a chimeric BTV-1/BTV-8 VP2 is replication competent and cross-reacts with BTV-1 and BTV-8 antisera. (A) Protein alignment of BTV-1 and BTV-8 VP2. (B) The plaque morphology of parental and chimeric synthetic reassortants [_B1_BTV-1^(BTV-8-220-429)^] is shown (left). Neutralization assays (right) of BTV-1, BTV-8, and _B1_BTV-1^(8-VP2-220-429)^ using BTV-1 and BTV-8 antisera were performed as described in Materials and Methods. BTV-1 and BTV-8 are neutralized only by homologous antisera. On the other hand, _B1_BTV-1^(8-VP2-220-429)^ is neutralized by both BTV-1 and BTV-8 antisera.

In addition to using the entire VP2 coding sequence of individual serotypes, we also tested whether it was possible to generate chimeric VP2 proteins cross-reacting with different serotypes. To achieve this, we designed a DNA plasmid encoding a chimeric VP2 protein in which amino acid residues from positions 220 to 429 of BTV-1 were substituted by the homologous region of BTV-8 VP2 (Fig. 3A). The design of this chimera was based on the presence of conserved residues in the BTV-1 and BTV-8 VP2 junction sites, while including divergent areas where neutralizing epitopes had been identified in previous studies (36–39). This novel VP2 segment was processed for reverse genetics and used in the successful rescue of a synthetic virus termed _B1_BTV-1^(8-VP2-220-429)^.

To determine whether the introduction of the BTV-8 VP2 region into the BTV-1 VP2 changed the antigenicity of the resulting chimeric proteins, we carried out virus neutralization assays of wild-type BTV-1, BTV-8, and _B1_BTV-1^(8-VP2-220-429)^ with sheep antisera raised against BTV-1 or BTV-8. Unlike parental viruses, which were neutralized only by the homologous antisera, _B1_BTV-1^(8-VP2-220-429)^ was cross-neutralized by both BTV-1 and BTV-8 antisera, suggesting that the design of “bivalent” synthetic viruses is theoretically feasible (Fig. 3B).

## DISCUSSION

In the present study we devised a novel vaccine platform for the production of inactivated synthetic viruses which facilitates vaccine production against current and newly emerging BTV serotypes. Importantly, the ability for these vaccines to be scaled to an industrial scale has been proven.

Our vaccine platform is based on the design and rescue of viruses containing the VP2 (or VP2 and VP5) of distinct BTV serotypes with a viral “backbone” already adapted to tissue culture conditions and vaccine production. We validated this approach by selecting two reassortants, _B1_BTV-8^VP2/VP5^ and _B1_BTV-8^VP2^, which have been prepared in an industrial setting and proved to protect sheep as effectively as current commercial inactivated vaccines based upon wild-type strains of BTV-8 (30).

**FIG 4:**
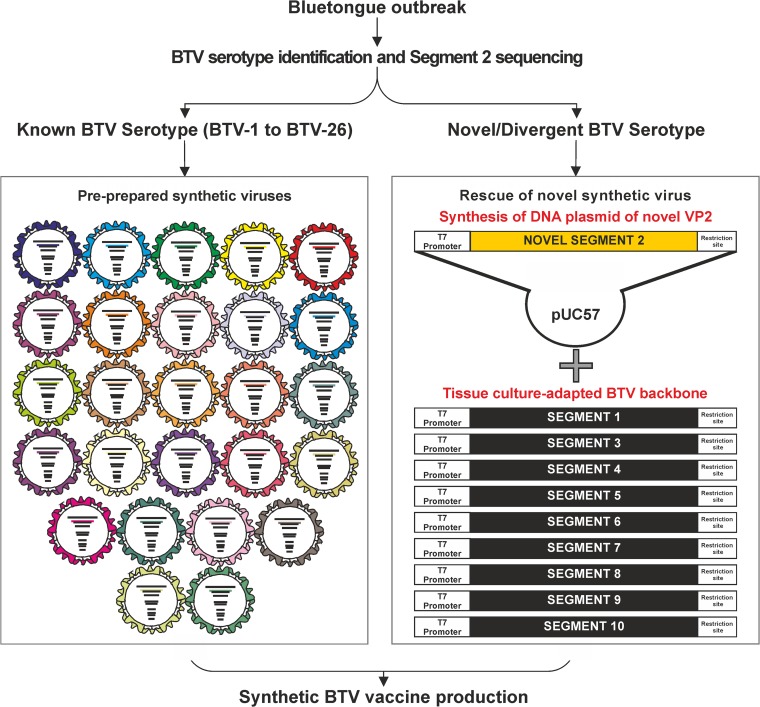
Vaccine platform based on inactivated synthetic viruses against current and emerging BTV serotypes. The strategy used for the development of a vaccine platform is based on synthetic reassortants formed by a “backbone” based on cell-culture-adapted BTV-1 and the outer core VP2 protein (or VP2 and VP5) of the serotype of interest. “Off-the-shelf“ or newly obtained vaccine strains will be used for vaccine development depending on the BTV strain involved in the outbreak to be contained.

If a new outbreak is caused by a currently known BTV serotype, pre-prepared “off-the-shelf” sBTV viruses, which have been validated for the initial steps of master-stock vaccine preparation, could be used in an accelerated vaccine production pipeline. In the case of an emerging BTV strain of a previously unknown serotype (or a particularly divergent strain within a known serotype), the nucleotide sequence of segment 2 (encoding the VP2 protein) is synthesized and cloned into a DNA plasmid vector which, in parallel with the tissue culture adapted BTV backbone, is used for the rescue of a replication competent sBTV virus by reverse genetics (Fig. 4). These vaccines are safe given that the inactivation step eliminates the risk of releasing replication competent pathogenic viruses into the environment. In addition, no secondary effects postvaccination were observed in any of the vaccinated animals in our trials.

The development of veterinary vaccines from discovery to regulatory approval and their use in the market is an extremely costly and lengthy process. Hence, vaccines for emerging diseases, especially if caused by pathogens with multiple distinct serotypes (and therefore requiring multiple products) are an especially big challenge for industry. In most cases, for obvious economic reasons, vaccine production initiates only after an outbreak occurs in an area where there is a potential market. In order to respond to BTV outbreaks, vaccine manufacturers have been able to significantly reduce the time needed to introduce new products into the market. However, it can take approximately 1 to 6 months for a vaccine manufacturer to acquire a new BTV strain from the field and a further 14 to 20 months to develop, test, produce a new vaccine and take it through provisional market authorization. These are conservative figures, and assume that every step runs smoothly, but it does happen that a seed vaccine strain may not pass all of the many steps required for vaccine development. Because all of the elements of the vaccines described here are derived synthetically, the acquisition of a viral isolate by industry and many of the quality control steps required by manufacturers prior to the introduction of field samples into the vaccine pipeline (e.g., presence of adventitious agents) can be bypassed and/or carried out more easily. In addition, a deeper understanding of possible barriers regulating reassortment of different genomic segments between specific BTV serotypes can also result in a further reduction in the time taken to obtain a new master seed for a new reassortant. Based upon current industry experience, the synthetic biology approach to vaccine development could save 6 months across the entire vaccine pipeline.

The timeline of vaccine production can be absolutely critical to halting the spread of a newly introduced BTV serotype. Most bluetongue outbreaks in temperate regions will have a limited diffusion in the first vector season after introduction but will spread considerably and cause extensive damage in the following year (40).

**FIG 5:**
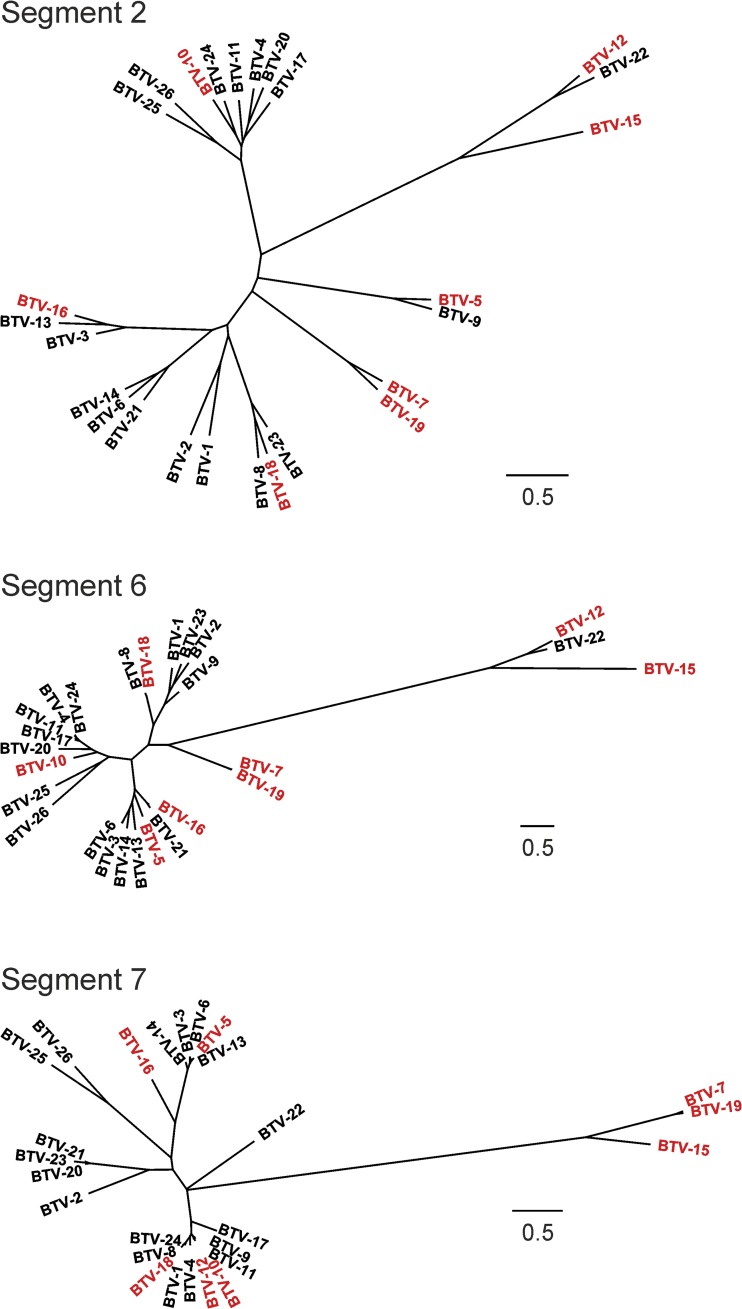
Phylogenetic relationships of the genomic segments encoding VP2, VP5, and VP7 of the BTV reference strains. Unrooted maximum-likelihood phylogenetic trees of the genomic segments encoding VP2, VP5, and VP7 of the BTV reference strains. Trees were estimated using PhyML (52) with 1,000 bootstraps under the best-fit model of nucleotide substitution determined using MODELTEST (53). Branch lengths are drawn to scale of nucleotide substitutions per site, with the bars representing 0.5 substitution/site. In red are indicated the serotypes for which we have not been able to rescue stable synthetic viruses in a BTV-1 backbone.

We have used and tested a single “backbone” in the current study based on BTV-1. It is likely that other backbones might be useful in order to increase the ability to rescue any BTV serotype/strain. We failed in the present study to obtain stable synthetic viruses containing the VP2 of BTV-5, -7, -10, -12, -15, -16, -18, -19. These serotypes do not seem to have any particular phylogenetic feature in common that could explain their unsuccessful rescue with a BTV-1 backbone (Fig. 5). Hence, more studies with additional backbones (or additional segment 2 sequences) will certainly shed light on the “compatibility” of different BTV proteins or genomic segments.

To further highlight the possibilities of genome manipulation for vaccine production, we also designed and rescued a synthetic BTV chimera containing a VP2 protein, including regions derived from both BTV-1 and BTV-8. Interestingly, while the parental viruses were neutralized only by homologous antisera, the chimeric proteins could be neutralized by both BTV-1 and BTV-8 antisera. These data suggest that neutralizing epitopes are present in different areas of the VP2 and likely “bivalent” strains eliciting neutralizing antibodies for multiple strains can be obtained. Thus, the potential exists to develop products made by two or three synthetic strains with chimeric VP2 that may be effective against multiple serotypes.

Several potential strategies for vaccination against BTV have been developed in the last decade (41). In mouse models, immunization with VP2 and VP5 expressed in bacteria has recently been shown to protect against lethal BTV challenge (42). Mice were also protected against BTV when VP2 was expressed in vectors such as bovine herpesvirus 4 (43), equine herpesvirus 1 (44), or modified vaccinia virus Ankara (45).

In sheep, virus-like particles derived using recombinant baculovirus expression systems have been shown to protect against virulent BTV challenge (46, 47). Vesicular stomatitis viral replicons expressing VP2 or similar vaccines based on canarypoxviruses have also been shown to be potentially effective vaccines for bluetongue (48, 49).

Vaccines based on reverse genetics include the generation of live attenuated vaccines in which the VP2 and VP5 proteins of BTV-1 and BTV-8 were introduced into an attenuated BTV-6 backbone (50). Disabled-infectious-single-cycle BTV vaccines have also proven to be effective in experimental trials in sheep. These vaccines are particularly interesting because they are generated by rescuing BTV in cell lines expressing one of the viral proteins in *trans* and therefore might possess most of the benefits of the live attenuated vaccines without their inherent risks (51).

However, the passage from a potential vaccine developed in an experimental setting to an industrial product ready to be used in the market is affected by several economic, scientific, and regulatory issues that are particularly complex for emerging diseases. The vaccine platform that we have developed has the advantage of utilizing synthetic biology in order to curtail the developmental period, while simultaneously equating to an existing commercial product and its known qualities. In turn, this allows the product to be rapidly introduced into the market when necessary.
